# Supplementary figures and images for: Pangenomic type III effector database of the plant pathogenic Ralstonia spp
Source: PeerJ. 2019 Aug 6;7:e7346. doi: 10.7717/peerj.7346 (PMC6762002; doi:10.7717/peerj.7346)

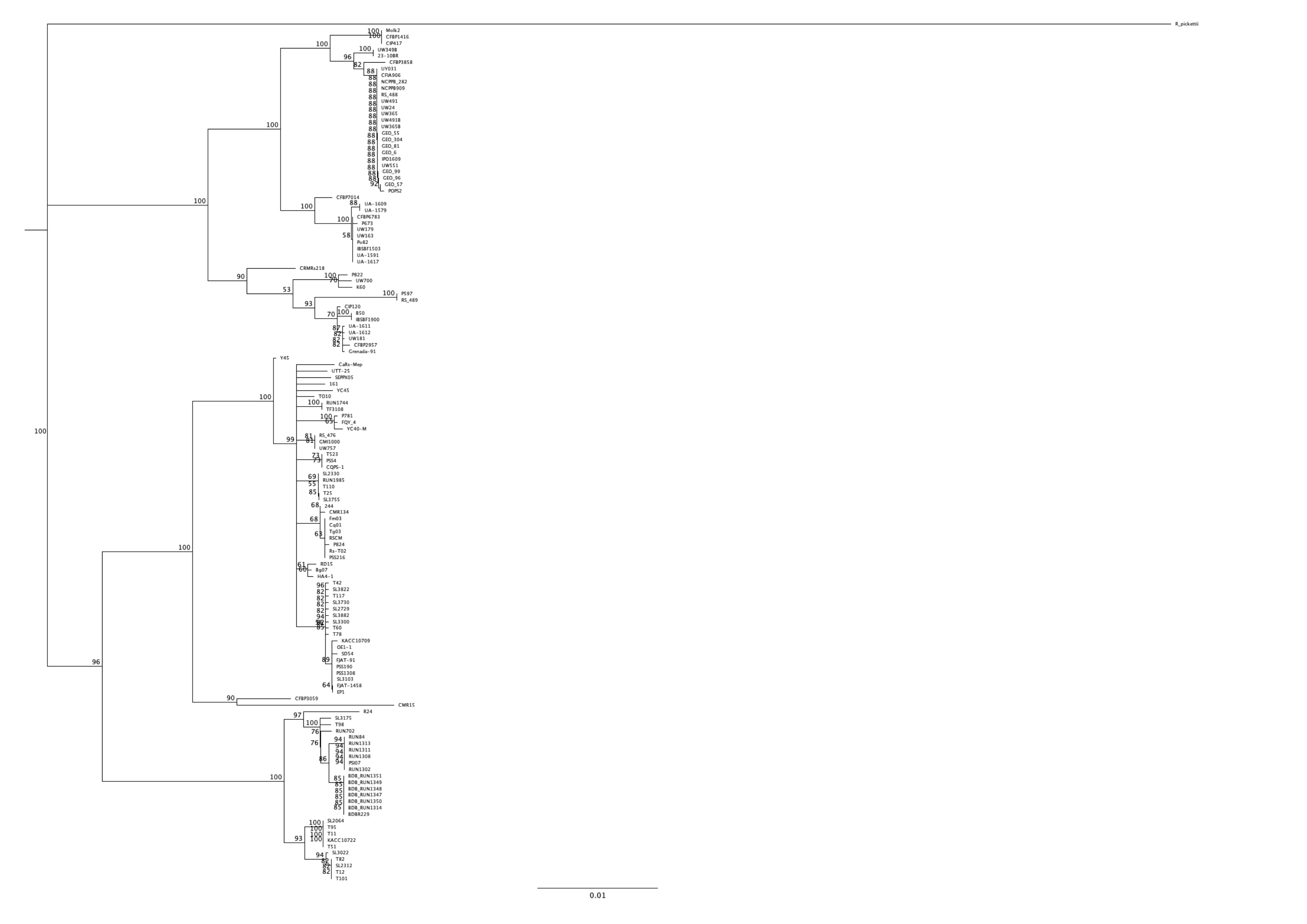

Supplement: Supplemental Information 2 — A neighbor-joining tree was build using the mutS from Ralstonia pickettii as an outgroup. Bootstrap were performed on 100 replicates, only support higher than 50% displayed in the consensus tree. [file peerj-07-7346-s002.png]
