# Supplementary material for: Pangenomic type III effector database of the plant pathogenic Ralstonia spp
Source: PeerJ. 2019 Aug 6;7:e7346. doi: 10.7717/peerj.7346 (PMC6762002; doi:10.7717/peerj.7346)

Pannel A

BACTERIAL PELLETS

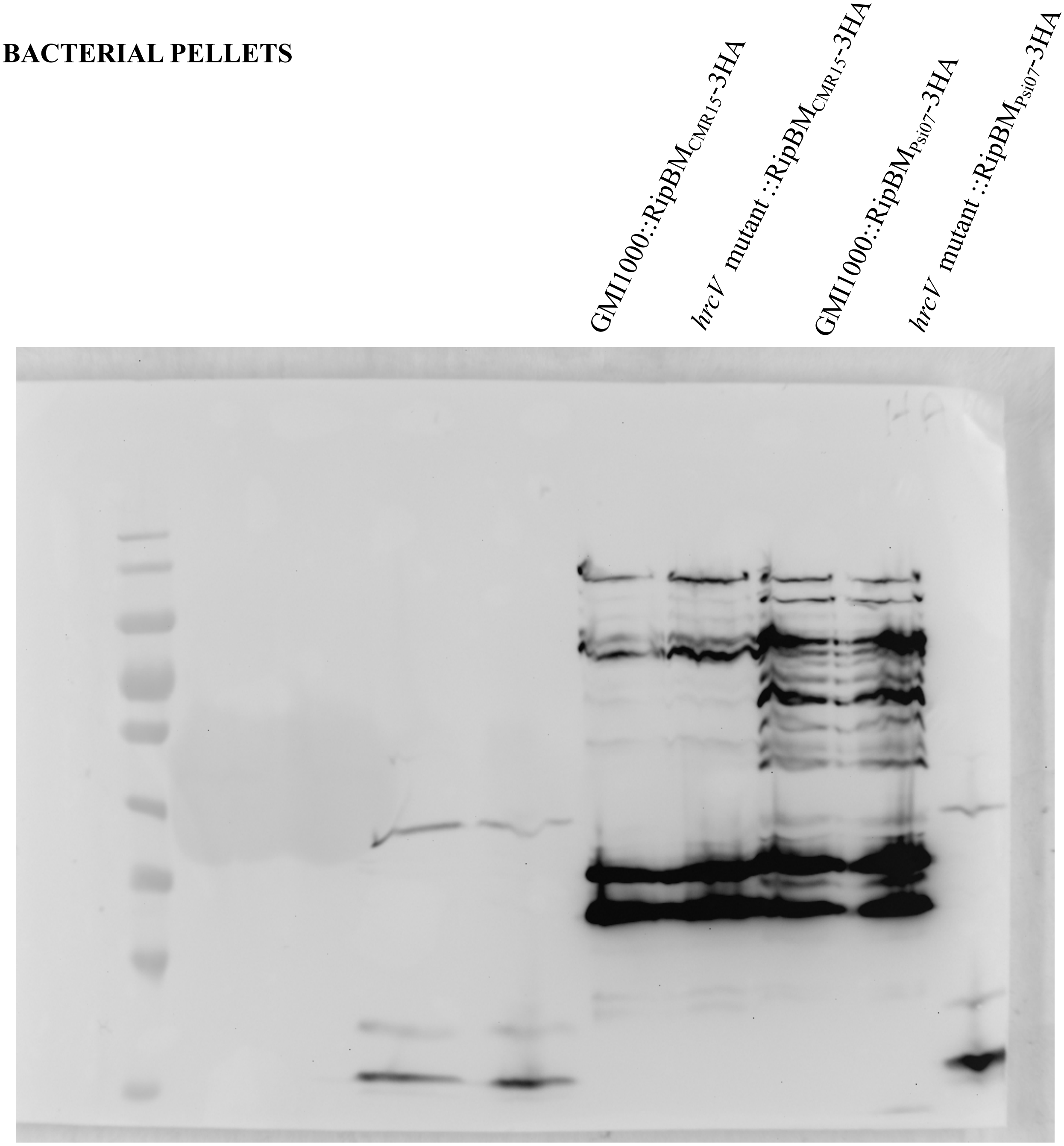

Pannel A

SECRETED PROTEINS

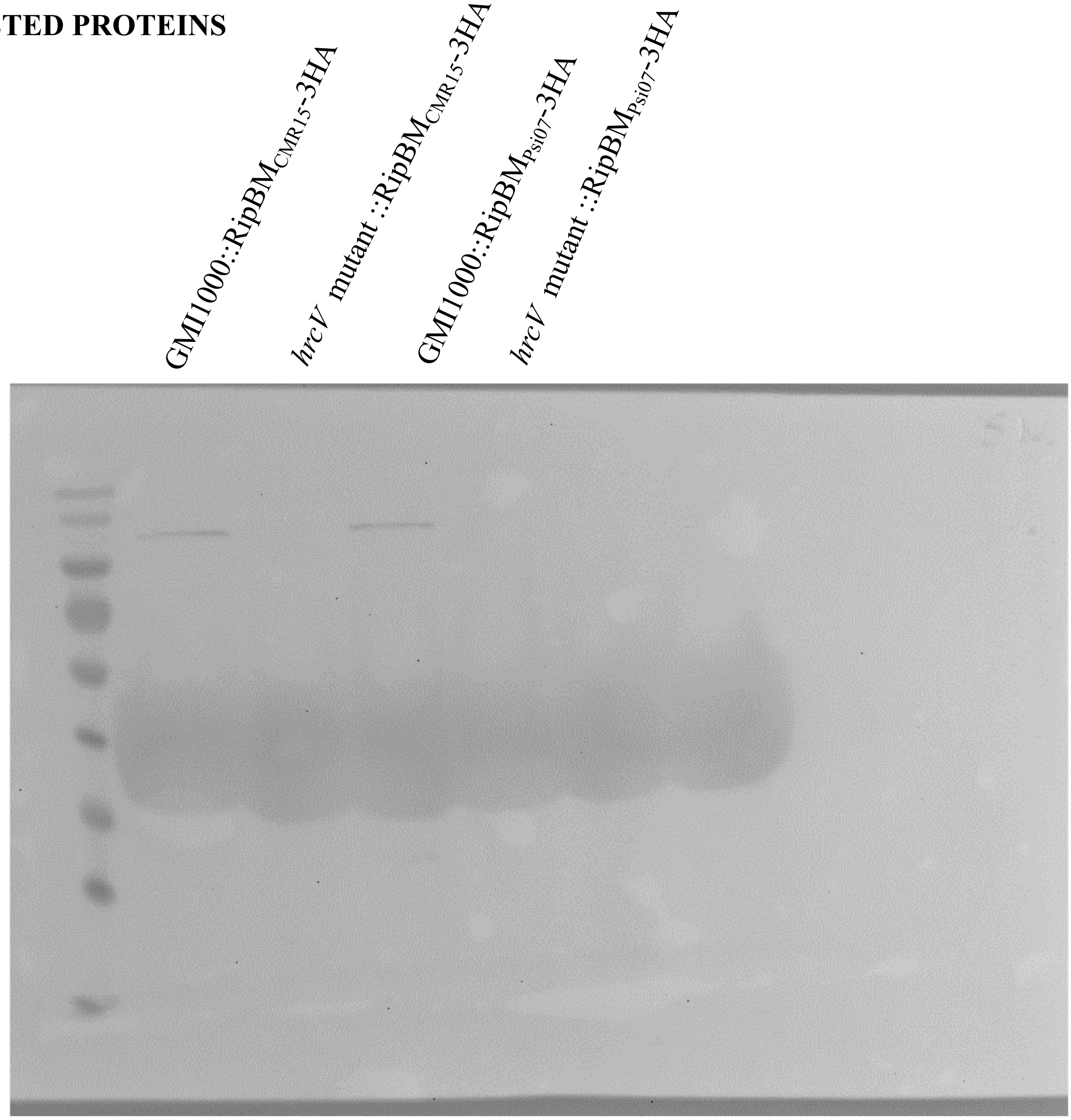

Pannel B

SECRETED PROTEINS **BACTERIAL PELLETS**

GMI1000::RipBO-3HA

hrcV mutant::RipBO-3HA

GMI1000::RipBO-3HA

hrcV mutant::RipBO-3HA

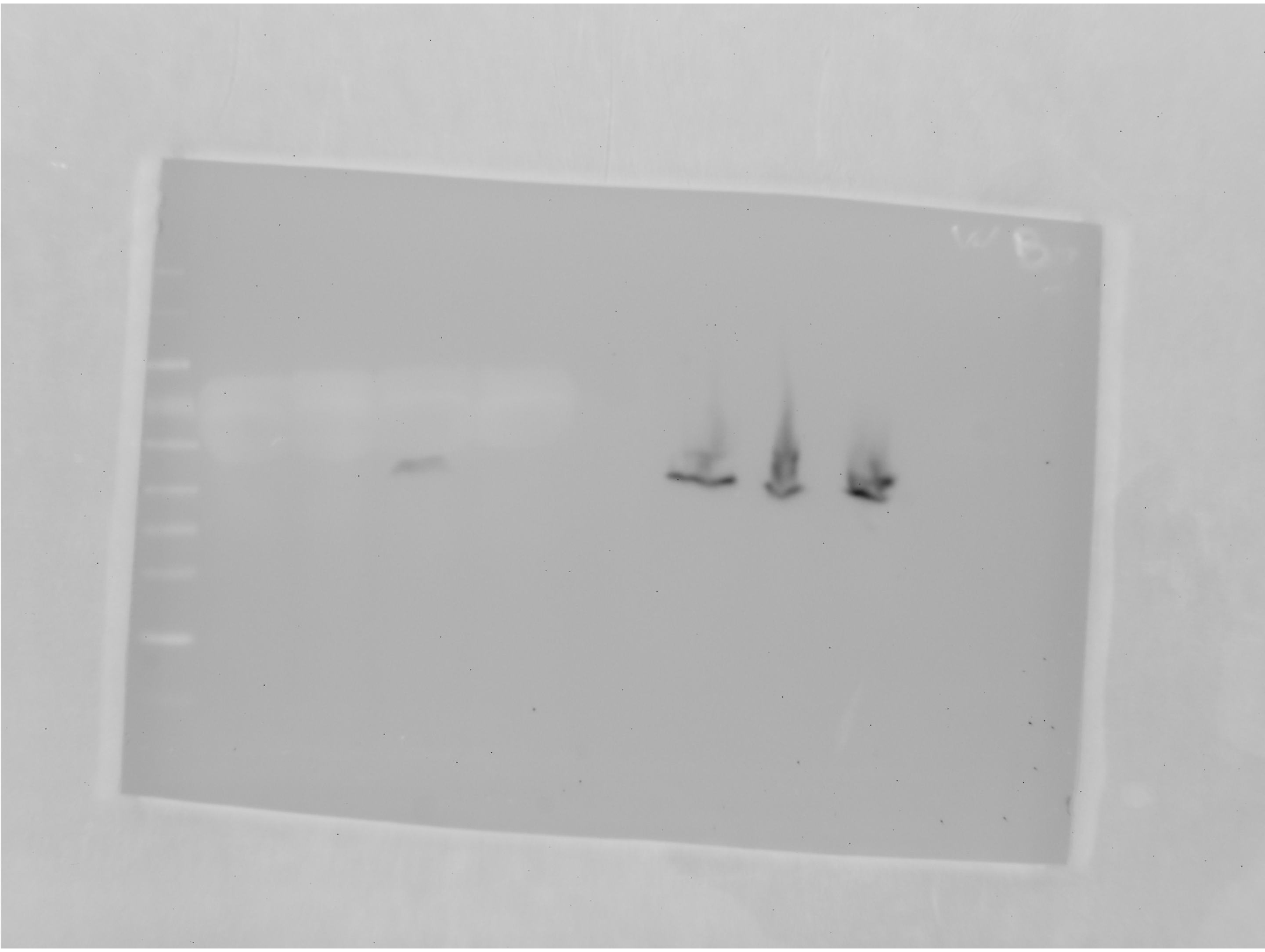

Supplement: Supplemental Information 3 [file peerj-07-7346-s003.pdf]
